# Prevalence and Factors Associated With Hypertension Among Type 2 Diabetic Patients in Private Health Facilities, Kampala, Uganda

**DOI:** 10.1155/ijhy/5298004

**Published:** 2026-05-18

**Authors:** Lesley Rose Ninsiima, Douglas Bulafu, Tom Okade, Evelyn Nakuya, James Natweta Baguma, Rogers Musiitwa, David Musoke

**Affiliations:** ^1^ Department of Disease Control and Environmental Health, Makerere University School of Public Health, Kampala, Uganda, musph.ac.ug; ^2^ Department of Biosecurity, Ecosystems and Veterinary Public Health, Makerere University, Kampala, Uganda, mak.ac.ug

**Keywords:** hypertension prevalence, private health facilities, Type 2 diabetes mellitus

## Abstract

**Introduction:**

Noncommunicable diseases (NCDs) such as Type 2 diabetes mellitus (T2D) and hypertension (HTN) are the leading causes of mortality worldwide, particularly in low‐ and middle‐income countries (LMICs). In Uganda, while private health facilities in urban areas are preferred for managing these conditions, there is limited information on the prevalence of HTN and associated risk factors among T2D patients in these settings. This study assessed the prevalence of HTN and associated factors among T2D patients in private health facilities, Kampala, Uganda.

**Methods:**

A health facility–based cross‐sectional study using quantitative methods was employed, which included obtaining information from the patient records and a structured questionnaire. The study recruited 295 participants at the diabetes clinics in selected private health facilities. The collected data were analysed using STATA 15.0 statistical software. Bivariate and multivariable analyses were conducted using a generalised linear model of modified Poisson regression.

**Results:**

Almost a third, 28.9% (85/295) of the participants had lived with T2D for more than 10 years. The prevalence of HTN among participants was 63.7%. Knowledge of HTN was low, with only 44.1% of the participants knowing about blood pressure, and 56.3% had a low level of knowledge overall. Participants who smoked (APR = 2.15, 95% Cl: 1.34–3.46), consumed an alcoholic drink in the past 30 days (APR = 1.56, 95% Cl: 1.10–2.20) and had a high level of knowledge on HTN (APR = 2.16, 95% Cl: 1.56–2.99) were more likely to have HTN.

**Conclusions:**

Our study found a worrying high burden of uncontrolled HTN among T2D patients, which could be due to risky behaviours including smoking and alcohol use. The generated evidence will inform feasible HTN risk reduction measures among T2D patients, which will improve awareness and existing controls set up by the different stakeholders. Therefore, there is a need for consolidated efforts aimed at addressing the identified gaps through community health education and lifestyle adjustments at facilities they attend to, especially for high‐risk behaviours such as smoking and alcohol use, to effectively reduce HTN prevalence in similar populations.

## 1. Introduction

Globally, noncommunicable diseases (NCDs) are one of the leading causes of mortality, with nearly three‐quarters of NCD‐related deaths occurring in low‐ and middle‐income countries (LMICs) [1]. Among the NCDs, Type 2 diabetes mellitus (T2D) has been found to coexist with hypertension (HTN), another leading component of the global burden of disease [2]. In sub‐Saharan Africa (SSA), the disease burden of both HTN and diabetes mellitus (DM) is anticipated to be twofold by 2025 and 2035, respectively [3]. The coexistence of HTN and DM ranges from 20% to 60%, varies with ethnicity, body mass index and age [4], and confers a dramatically increased risk (2‐ to 4‐fold) of cardiovascular disease, end‐stage kidney disease and death, compared with the normotensive and nondiabetic adults [5], thereby compromising public health.

In Uganda, measures have been put in place to curb the increasing NCD burden in the country, for example, the launch of the presidential initiative on healthy lifestyles and eating [6], launch of a National Day of Physical activity, which is held every year on the first Sunday of July to raise awareness on the increasing NCD burden in Uganda [7], development of an NCD Multi‐sectoral Strategic Plan [8], and others including restrictions on alcohol advertising and increasing alcohol taxes, control of tobacco use (advertising ban, tobacco taxes), trans‐fat and salt policies. Despite these efforts, a recent national NCD risk‐factor survey done in Uganda reported a high prevalence of HTN (> 25%) and alcohol use (> 20%) [9].

The majority of the T2D patients with HTN in Uganda living in urban areas such as Kampala obtain their medication from private healthcare facilities [10]. However, research on the capacity of private health facilities in Uganda has identified a number of barriers for HTN control, such as scarce medications and diagnostic equipment, insufficient knowledge and experience of clinicians in the management of HTN and T2D, and a lack of guidelines for HTN management [11]. These barriers have greatly limited the effectiveness of the private healthcare system in the management of HTN, especially among the T2D patients.

The available literature in Uganda among T2D concentrates mainly on the patient population’s coping strategies and difficulties [12, 13] but not comorbidities such as HTN. In addition, there is limited information on the prevalence of HTN and its associated risk factors among patients with T2D in private health facilities in Kampala [14]. Therefore, this study assessed the prevalence and factors associated with HTN among T2D patients in private health facilities in Kampala, Uganda.

## 2. Materials and Methods

### 2.1. Study Site

The study was conducted in private health facilities in Kampala district that manage NCDs, especially diabetic clinics. Most Ugandans in Kampala who had T2D preferred private health facilities due to easy access to health services, including prescription medications for T2D, HTN and HIV when indicated. The study was conducted in Nsambya, Mengo and Lubaga hospitals. Nsambya is a private not‐for‐profit hospital, located in the southern part of Kampala city, approximately 3 km from the city centre, and its service area is found in Makindye West subdistrict. Mengo is a private, faith‐based, community hospital located on Namirembe hill in Rubaga division, in northwestern Kampala, along Albert Cook Road. Lubaga is a private, not‐for‐profit, community hospital located on Lubaga Hill, in the Lubaga division, in the western part of Kampala. These hospitals had diabetic clinics which operated once a week and received about 50–60 patients each on the clinic days.

### 2.2. Study Design and Population

A cross‐sectional study using quantitative methods was employed. A structured questionnaire was used to collect quantitative data among T2D patients in the selected private health facilities in Kampala (supporting 1). T2D patients who had come to receive care at Lubaga, Mengo and Nsambya hospitals in Kampala during the study period met the eligibility criteria.

### 2.3. Sampling and Sample Size Determination

The sampling size of the study population was determined with the Leslie Kish formula, where *n* = the desirable sample size, (*α*/2) = the critical value at 95% level of significance (1.96), *p* = proportion of HTN among T2D (80%) [4], and *d* = precision of measurement (acceptable marginal error) (0.05). Considering *p*, prevalence of HTN among T2D patients = 80%, *N* = ((Zα/2)^2^ *p*(1 − *p*))/*d* ^2^, where the sample size for this study is 246. To achieve the sample size, we considered 16.6% nonresponse or dropout rate. Thus, when applying the formula: *N* = 246/(1 − 0.166) = 295.

Consecutive sampling was employed in this study. Respondents who came to the hospital were assessed for eligibility in their order of arrival at the clinic until the sample size was reached. From stakeholder engagements at these hospitals, they received approximately the same number of adult T2D patients (40–50 weekly). This implied that an equal number of participants from each of the three private health facilities, as illustrated below. Data were collected consecutively from these hospitals until the sample size was reached. Purposive sampling for the selection of the facilities was based on the patient enrolment for the T2D patients and treatment (Table 1).

**TABLE 1:** Number of participants chosen from each private health facility per week during the study period.

Hospital	Week 1	Week 2	Week 3	Week 4	Week 5	Total
Nsambya hospital	20	19	20	19	20	98
Mengo hospital	20	21	20	19	19	99
Lubaga hospital	20	19	20	19	20	98
Grand total						295

### 2.4. Data Collection

The study included T2D patients above 18 years on treatment for at least 3 months and attended diabetes care at Mengo, Lubaga and Nsambya hospitals during the study period. Patients who were critically ill, incapacitated or in any way unable to withstand study procedures were excluded from the study. Data were collected by trained research assistants with a background in health sciences and experience in collecting quantitative data. The study administered a structured questionnaire and reviewed patients’ medical records. The participants were recruited from 27^th^‐08‐2023 to 30^th^‐11‐2023.

The study defined the participants’ history of smoking as those who smoked at least one cigarette per day for at least 1 year. Patients who were critically ill, incapacitated or unable to withstand study procedures were excluded. Pregnant women were also excluded because pregnancy is associated with physiological changes in blood pressure and can lead to gestational HTN or pre‐eclampsia, which could confound the assessment of chronic HTN in T2D patients. The data were collected using a structured questionnaire. This standardised tool was adopted from the World Health Organization (WHO) Steps Instrument [15] to ensure systematic and comparable data collection. The tool was translated into Luganda, the commonly spoken language in Kampala. It was divided into various sections:

i. Sociodemographic characteristics like age, sex, marital status, employment status, educational level, private health facility, division (where the health facility is located), place of residence and where they came from to reach the health facility.
ii. Risk factors such as physical inactivity, tobacco smoking and alcohol consumption.
iii. Knowledge on HTN, such as signs and symptoms of high BP, HTN remedies.

### 2.5. Measurement of Study Variables

The outcome variable was having or not having HTN. To assess the outcome variable, a set of 9 questions was designed to assess whether patients had HTN or not (supporting 1). For each question, participants with a response ‘Yes/Always’ scored 1, while those with a response ‘No/Sometimes’ scored 0. A composite score was then generated based on the summation of all responses. The mean score was then used as the cut‐off. Participants who gained a total score above the mean were considered to have HTN, while those who obtained a total score equal to or below the mean were considered not to have HTN. Patients were classified as having HTN if they had a documented history of antihypertensive medication use. Additionally, their files/records were also checked to verify if they had HTN or not. Patients with systolic BP less than 140 mmHg and diastolic BP of less than 90 mmHg were classified as having normal BP (not having HTN). Considering the last three BP measurements taken as observed in their records/files, patients were classified as hypertensive if their mean systolic BP was 140 mmHg or greater, or if their average diastolic BP was 90 mmHg or higher [16]. These three measurements were taken across separate routine clinic visits by a health worker in the facility, which are usually spaced 1–4 weeks apart, depending on the patient’s appointment schedule at that facility [17]. In addition, those on HTN medication, as described by clinical records, were considered to have HTN.

The independent variables included sociodemographic characteristics (age, sex, marital status, employment status, educational level, private health facility, division, place of residence and where they came from to reach the health facility). Knowledge on HTN was assessed using 8 questions (supporting 1). Participants with a ‘yes’ response received one score, and 0 if otherwise. A composite score was then determined based on the total scores of correct responses. The mean score was then obtained and used as a cut‐off. Participants who obtained a total score above the mean were considered to have high knowledge, while those with a total score equal to or below the mean were considered to have low knowledge.

### 2.6. Data Management and Analysis

Quantitative data were collected using Android‐enabled mobile phones loaded with the Kobo Collect application software. The data were synchronised onto the server daily, and only the principal investigator and data manager had access to the server. The collected data were cleaned using Microsoft Excel 2016 and analysed using STATA 15.0 statistical software. Descriptive analyses such as frequencies, proportions and means (where appropriate) were performed for sociodemographic characteristics, knowledge on HTN and associated factors.

To assess the association between the outcome variable (having HTN or not) and each explanatory variable, we first performed bivariate analysis using a generalised linear model of modified Poisson regression, providing crude prevalence risk ratios (cPRs) and their corresponding 95% confidence intervals (CIs) as this method directly estimates prevalence ratios for common outcomes and is preferred over logistic regression in cross‐sectional studies. Variables with *p* < 0.2 were all added into the multivariable model to ascertain significant variables for each outcome. Also, factors with biological plausibility and those found significant in other studies were included in the multivariable model. This approach was chosen to adequately control for potential confounding while avoiding overadjustment. The statistical significance levels were two‐sided at *p* < 0.05.

## 3. Results

### 3.1. Sociodemographic Characteristics of T2D Patients in Private Health Facilities, Kampala

A total of 295 participants from three private health facilities participated in the study, with the majority, 63.0% (186/295), attending Nsambya hospital for treatment, and 68.1% (201/295) were females. More than half, 52.5% (155/295) of the participants were aged 60 years and above, with a mean age of 59.3 years (SD 11.8). Less than half, 35.9% (106/295) of the participants had attained primary education, and 51.2% (190/295). More than half, 55.6% (164/295) of the participants came from within Kampala to reach the health facility (Table 2).

**TABLE 2:** Sociodemographic characteristics of T2D patients in private health facilities, Kampala.

Variable	Frequency (*N* = 295)	Percentage (%)
*Private health facility*		
Lubaga hospital	43	14.6
Mengo hospital	66	22.4
Nsambya hospital	186	63.0

*Division*		
Makindye	188	63.7
Lubaga	107	36.3

*Sex*		
Male	94	31.9
Female	201	68.1

*Age (years)*	*Mean (SD) = 59.3 ± 11.8*	
< 60	140	47.5
≥ 60	155	52.5

*Marital status*		
Divorced/separated	18	6.1
Married/cohabiting	172	58.3
Single	37	12.5
Widowed	68	23.1

*Highest academic qualification*		
Never attended	22	7.5
Primary	106	35.9
Secondary	105	35.6
Tertiary	62	21.0

*Employment status*		
Farmers	37	12.5
Formally employed/civil servants	40	13.6
Informally employed	32	10.9
Self‐employed/business/traders	96	32.5
Unemployed	90	30.5

*Place of residence*		
Rural area	151	51.2
Urban area	144	48.8

*Where they came from to reach the health facility*		
Outside Kampala	131	44.4
Within Kampala (one of the divisions of KCCA)	164	55.6

Abbreviation: SD, standard deviation.

### 3.2. Risk Factors of HTN Among T2D Patients in Private Health Facilities, Kampala

Among the participants, 2.7% (8/295) smoked, and 10.9% (32/295) used alcohol in the past 30 days. Less than half, 49.5% (146/295) of the participants did not do any physical activity in the past 7 days. However, 53.9% (159/295) of the participants walked for more than 5 days. Almost a third, 28.9% (85/295) of the participants had lived with T2D for more than 10 years (Table 3).

**TABLE 3:** Risk factors of hypertension among T2D patients in private health facilities, Kampala.

Variable	Frequency (*N* = 295)	Percentage (%)
*Smoking status*		
No	287	97.3
Yes	8	2.7

*Frequency of smoking*	*(n = 8)*	
Always	2	25.0
Sometimes	6	75.0

What was smoked*^∗^*	*(n = 8)*	
Hand‐rolled cigarettes	2	25.0
Manufactured cigarettes	2	25.0
Pipes full of tobacco	4	50.0
Shisha	1	12.5

*Days someone smoked around them*		
< 1	258	87.5
≥ 1	37	12.5

*Alcohol use*		
No	263	89.1
Yes	32	10.9

*Number of occasions of at least one alcohol in the past 30 days*	*(n = 32)*	
< 5	21	65.6
≥ 5	11	34.4

*Days of physical activity in the past 7 days*		
0	146	49.5
1–4	88	29.8
> 4	61	20.7

*Days of walking in the past 7 days*		
≤ 5	136	46.1
> 5	159	53.9

*Years lived with diabetes*		
≤ 10	209	71.1
> 10	85	28.9

^∗^Multiple response.

### 3.3. Type 2 Diabetic Patients’ Knowledge on HTN

Less than half, 44.1% (130/295) of the participants knew something about BP, and of these, 55.4% (72/130) of the participants mentioned that the normal BP level is between 90/60 mmHg and 120/80 mmHg. Among the 130 participants who knew about BP, the majority, 66.9% (87/130), reported early morning headaches as a sign and symptom of high BP, and 56.9% (74/130) reported chest pain as a complication of high BP. Overall, more than half, 56.3% (166/295) of the participants had low knowledge on HTN (Table 4).

**TABLE 4:** Type 2 diabetic patients’ knowledge on hypertension.

Variable	Frequency (*N* = 295)	Percentage (%)
*Knew anything about blood pressure*		
No	165	55.9
Yes	130	44.1

*Normal blood pressure level in mmHg*	*(n = 130)*	
Above 120/180	11	8.4
Below 90/60	1	0.8
Between 90/60 and 120/80	72	55.4
Did not know	46	35.4

Signs and symptoms of high blood pressure*^∗^*	*(n = 130)*	
Early morning headaches	87	66.9
Nose bleeds	7	5.4
Irregular heart rhythms	53	40.8
Vision changes	44	33.9
Buzzing in the ears	3	2.3
Nausea	15	11.5
Vomiting	5	3.9
Confusion	9	6.9
Anxiety	38	29.2
Chest pain and muscle tremors	47	36.2

Complications of high blood pressure*^∗^*	*(n = 130)*	
Chest pain	74	56.9
Heart attack	42	32.3
Heart failure	26	20.0
Irregular heartbeat	50	38.5
Kidney damage	50	38.5

Hypertension treatment options*^∗^*	*(n = 130)*	
Water pills (diuretics)	11	8.5
Angiotensin‐converting enzyme (ACE) inhibitors	7	5.4
Angiotensin receptor blockers (ARBs)	3	2.3
Calcium channel blockers	13	10.0
Others (e.g., Arbiter‐H, aspirin)	2	1.5
Don’t know	105	80.8

*Knew other hypertension treatment remedies*	*(n = 130)*	
No	104	80.0
Yes	26	20.0

*Treatment remedies*	*(n = 26)*	
Avoid anxiety	3	11.5
Healthy diet	3	11.5
Herbal medicine	12	46.2
Regular exercise	8	30.8

Risk factors of hypertension*^∗^*	*(n = 130)*	
Unhealthy diet	77	59.2
Physical inactivity	98	75.4
Obesity/overweight	42	32.3
Family history of hypertension	35	26.9
Age	58	44.6
Consumption of alcohol and tobacco	16	12.3
Coexisting diseases	20	15.4
Stress and anxiety	23	17.7

*Level of knowledge on HTN*		
High knowledge	129	43.7
Low knowledge	166	56.3

^∗^Multiple response.

### 3.4. Factors Associated With HTN Among T2D Patients in Private Health Facilities, Kampala

The prevalence of HTN among the participants was 63.7% (188/295). At bivariate analysis, sex, division, private health facility, age, employment status, place of residence during childhood, smoking status, alcohol use and level of knowledge on HTN were associated with having HTN. After adjusting for potential confounders, smoking, consuming alcohol in the past 30 days and having a high level of knowledge on HTN were significantly associated with having HTN. Participants who smoked were 2.1 times more likely to have HTN when compared with those who did not smoke (APR = 2.15, 95% Cl: 1.34–3.46). Participants who consumed an alcoholic drink in the past 30 days were 1.5 times more likely to have HTN as compared to their counterparts that did not consume alcohol (APR = 1.56, 95% Cl: 1.10–2.20). When compared to those who had a low level of knowledge on HTN, participants who had a high level of knowledge on HTN were 2.1 times more likely to have HTN (APR = 2.16, 95% Cl: 1.56–2.99). However, sex was not significant at multivariable analysis (Table 5).

**TABLE 5:** Factors associated with hypertension among T2D patients in private health facilities, Kampala.

**Variable**	**Attribute**	**Hypertension status**		**Crude PR (95% Cl)**	**p** **values**	**Adjusted PR (95% Cl)**	**p** **values**
		**Yes (*n* = 188)**	**No (*n* = 107)**				

Sex	Female	133 (66.2)	68 (33.8)	1		1	
	Male	55 (58.5)	39 (41.5)	1.23 (0.90–1.67)	**0.195**	0.92 (0.66–1.30)	0.639

Division	Makindye	105 (55.9)	83 (44.1)	1			
	Rubaga	83 (77.6)	24 (22.4)	0.51 (0.34–0.75)	**0.001**		

Private health facility	Lubaga	35 (81.4)	8 (18.6)	1			
	Mengo	47 (71.2)	19 (28.8)	1.55 (0.74–3.22)	0.243		
	Nsambya	106 (57.0)	80 (43.0)	2.31 (1.21–4.42)	**0.011**		

Age (years)	< 60	83 (59.3)	57 (40.7)	1		1	
	≥ 60	105 (67.7)	50 (32.3)	0.79 (0.58–1.07)	**0.133**	0.85 (0.63–1.14)	0.286

Marital status	Divorced/separated	12 (66.7)	6 (33.3)	1			
	Married/cohabiting	104 (60.5)	68 (39.5)	1.19 (0.60–2.34)	0.623		
	Single	24 (64.9)	13 (35.1)	1.05 (0.48–2.32)	0.896		
	Widowed	48 (70.6)	20 (29.4)	0.88 (0.42–1.87)	0.744		

Highest academic qualification	Never attended	16 (72.7)	6 (27.3)	1			
	Primary	63 (59.4)	43 (40.6)	1.49 (0.72–3.06)	0.281		
	Secondary	73 (69.5)	32 (30.5)	1.12 (0.53–2.35)	0.769		
	Tertiary	36 (58.1)	26 (41.9)	1.54 (0.73–3.24)	0.257		

Employment status	Farmers	21 (56.8)	16 (43.2)	1	1	1	
	Formally employed	25 (62.5)	15 (37.5)	0.87 (0.50–1.50)	0.609	0.69 (0.39–1.20)	0.189
	Informally employed	18 (56.3)	14 (43.7)	1.01 (0.59–1.74)	0.966	0.95 (0.56–1.63)	0.865
	Self‐employed	59 (61.5)	37 (38.5)	0.89 (0.57–1.40)	0.615	0.78 (0.49–1.24)	0.289
	Unemployed	65 (72.2)	25 (27.8)	0.64 (0.39–1.06)	**0.082**	0.66 (0.41–1.06)	0.088

Place of residence	Rural area	105 (69.5)	46 (30.5)	1		1	
	Urban area	83 (57.6)	61 (42.4)	1.39 (1.02–1.89)	**0.036**	0.065	1.33

Where they came from to reach the health facility	Outside Kampala	80 (61.1)	51 (38.9)	1			
	Within Kampala (one of the divisions of KCCA)	108 (65.9)	56 (34.1)	0.88 (0.65–1.19)	0.395		

Smoking status	No	187 (65.2)	100 (34.8)	1		1	
	Yes	1 (12.5)	7 (87.5)	2.51 (1.85–3.41)	**< 0.001**	2.15 (1.34–3.46)	**0.001**

Alcohol use	No	176 (66.9)	87 (33.1)	1		1	
	Yes	12 (37.5)	20 (62.5)	1.89 (1.37–2.60)	**< 0.001**	1.56 (1.10–2.20)	**0.012**

Level of knowledge on HTN	Low	127 (76.5)	39 (23.5)	1		1	
	High	61 (47.3)	68 (52.7)	2.24 (1.63–3.09)	**< 0.001**	2.16 (1.56–2.99)	**< 0.001**

*Note:* Statistical significance = *p* < 0.25^∗^, *p* < 0.05^∗∗^.

## 4. Discussion

This study established a high burden of uncontrolled HTN, detected in three private health facilities of HTN patients with concomitant T2D, in Kampala, Uganda. This study found a high prevalence of HTN, 63.7%, among T2D patients. This is similar to other studies done in Uganda, which found a high prevalence of HTN among T2D patients [18, 19]. Therefore, these findings imply that there is an increase in the number of patients with uncontrolled HTN in the recent years. Thus, healthcare facilities should prioritise regular and routine screening for all patients regardless of their current HTN status, to enhance early detection and reduce further complications. Furthermore, home‐to‐home blood pressure screening programs should be strengthened and made affordable to the poor population, including patient saving groups, to buy their own BP machines to enhance regular BP monitoring. For proper management of HTN among patients with T2D, early evaluation for HTN and periodic evaluation of patients with pre‐HTN, applying lifestyle modifications and prompt treatment of HTN and hyperglycaemia will enable a good outcome [20]. The study also saw an imbalance in the patient flows despite the initial expectations because of the differences in actual patient attendance, such as variations in clinic days, temporary reductions in patient turnout at two sites due to seasonal factors and competing health campaigns. Although we employed consecutive sampling at all sites simultaneously until the overall target sample size was reached, one facility consistently had higher daily attendance during the recruitment window, leading to a faster accumulation of eligible participants.

Several lifestyle factors, including smoking and alcohol use, were associated with HTN as reported in this study. Similarly, a previous study reported a higher risk of HTN among smokers as compared to nonsmokers [21]. Alcohol consumption was more prevalent, with 10.9% of participants reporting alcohol use in the past 30 days. The relationship between alcohol use and HTN was statistically significant, suggesting that alcohol consumption may increase the risk of HTN in this population. These findings are similar to those from other studies done in SSA [22, 23]. Health education campaigns on lifestyle risk factors (smoking and alcohol use) and management of HTN for high‐risk patients, particularly those with T2D, should be implemented by health professionals at these private health facilities.

The study found that participants who had a high level of knowledge on HTN were more likely to have HTN compared to those who had a low level of knowledge on HTN. However, a study done in Uganda reported that having knowledge on HTN was related to making informed decisions on lifestyle measures, reducing the prevalence of HTN [24]. This study’s findings may be affected by factors, including reverse causality, where the T2D patients acquire knowledge after being diagnosed with HTN, or confounding related to healthcare exposure, whereby T2D patients at a severe stage of the disease or those who have frequent clinic visits receive more health education than the non‐T2D patients. Therefore, additional nationwide surveys are needed to unravel the reason for this discrepancy. To reduce the risk of HTN among T2D patients, efforts to mitigate the possible impacts should consider integrative measures, which include increasing awareness and intensifying routine screening to improve overall health outcomes. The study’s findings implore private health facilities to increase utilisation of health education and lifestyle interventions such as regular physical activity for patients before they receive their medications and follow‐up of T2D patients for tight control of their blood pressure.

In the analysis, we did not find an association between HTN and some of the common factors that have often been reported to be associated with HTN, including sex [25] and age [26], which implies that there could be other risk factors that are crucial and relevant for HTN in the Ugandan setting, other than those analysed in this study. Therefore, broader investigative research on HTN and T2D is required to explore other factors that this study may not have explored.

This study had strengths and limitations. We adopted a robust methodology by employing a standardised tool (WHO Steps Instrument) for data collection, which ensured systematic and comparable data collection, enhancing the credibility of the findings. This study was conducted in only three private health facilities in Kampala, Uganda, which limits the generalisability of our findings to broader populations or diverse settings with distinct characteristics in Uganda. We used a relatively inclusive multivariable model to control for multiple potential confounders. While this approach reduces residual confounding, the model is not highly parsimonious. Some variables retained in the final model were no longer statistically significant after adjustment. We acknowledge this as a limitation; however, effect estimates for the main significant associations (smoking, recent alcohol use and high knowledge on HTN) remained stable when less parsimonious variables were removed. Future studies with larger samples may benefit from more refined model selection techniques or penalised regression approaches. Nonetheless, our study contributes to the understanding of HTN in T2D patients, providing valuable insights for tailored healthcare interventions and informing public health strategies.

## 5. Conclusion

Our study found a worrying high burden of uncontrolled HTN among T2D patients in the three private health facilities of Kampala district. Smoking and alcohol use were associated with higher HTN risk, underscoring the need for lifestyle‐focused interventions. Low awareness about HTN and misconceptions regarding normal BP levels reflect critical knowledge gaps, suggesting that preventive education is essential. Addressing these gaps through community health education and lifestyle adjustments at the facilities they attend to, especially for high‐risk behaviours such as smoking and alcohol use, could reduce HTN prevalence in similar populations.

## Author Contributions

Lesley Rose Ninsiima, Douglas Bulafu and Tom Okade supported the conceptualisation of the study and the development of data collection tools. Lesley Rose Ninsiima, Evelyn Nakuya, James Natweta Baguma, Tom Okade and Rogers Musiitwa collected the data and helped in data analysis. David Musoke was supported in the supervision and mentorship. However, Lesley Rose Ninsiima takes full responsibility for the conduct of the study and final manuscript as the guarantor; she had full access to the data and controlled the decision to publish.

## Funding

This study was funded by the Royal Society of Tropical Medicine and Hygiene (Grant 2022) (RSTMH) through the National Institute for Health and Care Research (NIHR).

## Disclosure

All authors contributed to the article and approved the submitted version. All authors reviewed and approved the final manuscript.

## Ethics Statement

Research ethical approval to conduct the study was obtained from the Makerere University School of Public Health Research and Ethics Committee (MakSPH‐REC: SPH 2023‐379). The study was also registered by the Uganda National Council for Science and Technology (Ref No: HS2828ES). Administrative clearance was obtained from Kampala City Council Authority (KCCA) and the hospital administration. After clearly explaining the study to the participants, written informed consent was obtained from them before commencing data collection and their names were not recorded. The data collected were stored on drives that were only accessed by the principal investigator and data manager.

## Conflicts of Interest

The authors declare no conflicts of interest.

## Supporting Information

Additional supporting information can be found online in the Supporting Information section.

## Supporting information


**Supporting Information** supporting 1—Quantitative data collection tool.

## Data Availability

The data that support the findings of this study are available on request from the corresponding author. The data are not publicly available due to privacy or ethical restrictions.
