# Supplementary material for: Prevalence and Factors Associated With Hypertension Among Type 2 Diabetic Patients in Private Health Facilities, Kampala, Uganda
Source: Int J Hypertens. 2026 May 18;2026:5298004. doi: 10.1155/ijhy/5298004 (PMC13181425; doi:10.1155/ijhy/5298004)
Supplement: Supplementary file 1 — Supporting Information supporting 1—Quantitative data collection tool. [file IJHY-2026-5298004-s001.docx]

**Supplementary 1**

Prevalence and Factors Associated with Hypertension among Type 2 Diabetic Patients in Private Health Facilities, Kampala, Uganda

**QUANTITATIVE DATA COLLECTION TOOL - QUESTIONNAIRE**

| **NO** | **QUESTION** | **RESPONSE** |
| --- | --- | --- |
|  | **SECTION A: SOCIO-DEMOGRAPHIC CHARACTERITICS** | |
|  | **Patient characteristics (Circle what applies)** | |
| **A1** | Private Health  facility………………………………………………………………………….  Location**…………………………………………………………………………**  **……………………. (Capture GPS)** | |
| **A2** | Interviewer ID  …………………………………………………………………………………… | |
| **A3** | Division | 1. Nakawa 2. Makindye 3. Kawempe 4. Central 5. Rubaga |
| **A5** | Sex of respondent | 1. Male 2. Female |
| **A6** | Age of patient in complete years | ………... Years |
| **A7** | Marital status of patient | 1. Single 2. Married/cohabiting 3. Widowed 4. Divorced/separated |
| **A8** | Highest Academic Qualification (Write the level of education even if it was completed e.g. a participant who ended in S.3 is at O level) | 1. Never attended 2. O level 3. A level 4. Tertiary 5. University |
| **A9** | Employment Status | 1. Employed 2. Self employed 3. Housewife 4. Unemployed 5. Casual business e.g. chapatti stall |
| **A10** | Where did you live for most of your childhood life (below 18 years)? | 1. Rural area 2. Urban area |
| **A11** | Where have you come from to reach this health facility? | 1. Within Kampala (one of the divisions of KCCA) 2. Outside Kampala |

|  | **SECTION B: RISK FACTORS**  **READ ALOUD:** *I am going to ask you some questions about various health indicators such as smoking, drinking alcohol, physical activity and quality of life.*  *Let us start with tobacco use* | |
| --- | --- | --- |
|  | **TOBACCO USE** |  |
| **B1** | Do you currently smoke any tobacco products such as cigarettes, cigars, pipes? | 1. Yes 2. No |
| **B2** | If yes, how often do you smoke any tobacco products? | 1. Always 2. Sometimes |
| **B3** | Which of the following do you smoke daily? (tick all mentioned)? | 1. Manufactured cigarettes 2. Hand rolled cigarettes 3. Pipes full of tobacco 4. Shisha |
| **B4** | In the past 7 days, on how days did someone smoke in your home when you were present? | _________  Don’t know999 |
|  | **ALCOHOL USE** |  |
| **B5** | Have you consumed an alcoholic drink in the past 30 days? | 1. Yes 2. No |
| **B6** | During the past 30 days, on how many **occasions** did you have at least one alcoholic drink? 999 Don’t know (*Occasion means seatings. The number of times one seats to take at least one alcoholic drink*) ***PROBE TO EXPLAIN this to the respondent for them to understand*** | ……………………. |
|  | **PHYSICAL ACTIVITY:**  **Vigorous physical** activities make you breathe much harder than normal physical activity and may include *lifting, digging, fast bicycling, playing football, and related games etc.* **Moderate physical** activities don’t make you breathe as hard as the vigorous. Think about activities you do at work, at home and during leisure time | |
| **B7** | During the past 7 days, on how many days did you do vigorous physical activity?  (Activities are called activities when done for at least 10 minutes in a row) | ……………………………. |
| **B8** | During the past 7 days, how many days did you do moderate physical activity?  (Activities are called activities when done for at least 10 minutes in a row) | …………………………. |
| **B9** | How long have you been living with type 2 diabetes? | …………………………….. |

|  | **SECTION C: TYPE 2 DIABETIC PATIENTS’ KNOWLEDGE ON HYPERTENSION** |  |
| --- | --- | --- |
| **C1** | Do you know anything about blood pressure? | 1. Yes 2. No……. |
| **C2** | What is the normal blood pressure level? | 1. Below 90/60 mmHg 2. Between 90/60 mmHg and 120/80 mmHg 3. Above 120/80 mmHg |
| **C3** | What are some of the signs and symptoms of high blood pressure? | 1. Early morning headaches 2. Nose bleeds 3. Irregular heart rhythms 4. Vision changes 5. Buzzing in the ears 6. Nausea 7. Vomiting 8. Confusion 9. Anxiety 10. Chest pain and muscle tremors 11. Others (specify) |
| **C4** | What are the complications of high blood pressure? | 1. Chest pain 2. Heart attack 3. Heart failure 4. Irregular heartbeat. 5. Kidney damage |
| **C5** | What are some of the hypertension treatment options you know? | 1. Water pills (diuretics) 2. Angiotensin-converting enzyme inhibitors (ACE) 3. Angiotensin II receptor blockers (ARBs) 4. Calcium channel blockers. 5. Other ………………………………. (please specify) |
| **C6** | Are there any other hypertension treatment remedies that you know? | 1. Yes 2. No |
| **C7** | If yes, which treatment remedies do you know? | ……………………………………… (please specify) |
| **C8** | What do you think are some of the risk factors of hypertension? | 1. Unhealthy diets 2. Physical inactivity 3. Consumption of Tobacco and alcohol 4. Being overweight/obese 5. Family history of hypertension 6. Age 7. Co-existing diseases. |

|  | Recent reading of blood pressure. (*If respondent found in the health centre, ask for their book or receipt to note down the recently measured blood pressure level.*  *If they remember the measurement, they can tell you what it was*) | ……………….. |
| --- | --- | --- |
